# Unravelling 3D growth in the moss *Physcomitrium patens*

**DOI:** 10.1042/EBC20220048

**Published:** 2022-12-08

**Authors:** Laura A. Moody

**Affiliations:** Department of Biology, University of Oxford, South Parks Road, Oxford, OX1 3RB, U.K.

**Keywords:** 3D growth, developmental biology, genetics

## Abstract

The colonization of land by plants, and the greening of the terrestrial biosphere, was one of the most important events in the history of life on Earth. The transition of plants from water to land was accompanied, and largely facilitated, by the acquisition of apical cells with three or more cutting faces (3D growth). This enabled plants to develop the morphological characteristics required to survive and reproduce effectively on land and to colonize progressively drier habitats. Most plants develop in such a way that makes genetic studies of 3D growth difficult as the onset of 3D growth is established early during embryo development. On the other hand, in the moss *Physcomitrium patens*, the onset of 3D growth is preceded by a protracted 2D filamentous phase of the life cycle that can be continuously propagated. *P. patens* is an ideal model system in which to identify the genetic toolkit underpinning the 2D to 3D growth transition, and this is because 3D growth is not a pre-requisite for survival. Thus, insights into the mechanisms underpinning the formation of apical cells and the subsequent establishment and maintenance of 3D growth have largely been gained through studies in *P. patens*. This review summarizes the most recently published articles that have provided new and important insights into the mechanisms underpinning 3D growth in *P. patens*.

## Introduction

One of the most revolutionary events in history was the colonization of land by plants around 470 million years ago. The transition from water to land coincided with, and was largely enabled by, the emergence of 3D growth, which created new opportunities for land plant diversification [1,2]. 3D growth is a pivotal and essential feature of all land plants and underpins the morphological diversity that shapes our planet.

**Figure 1:**
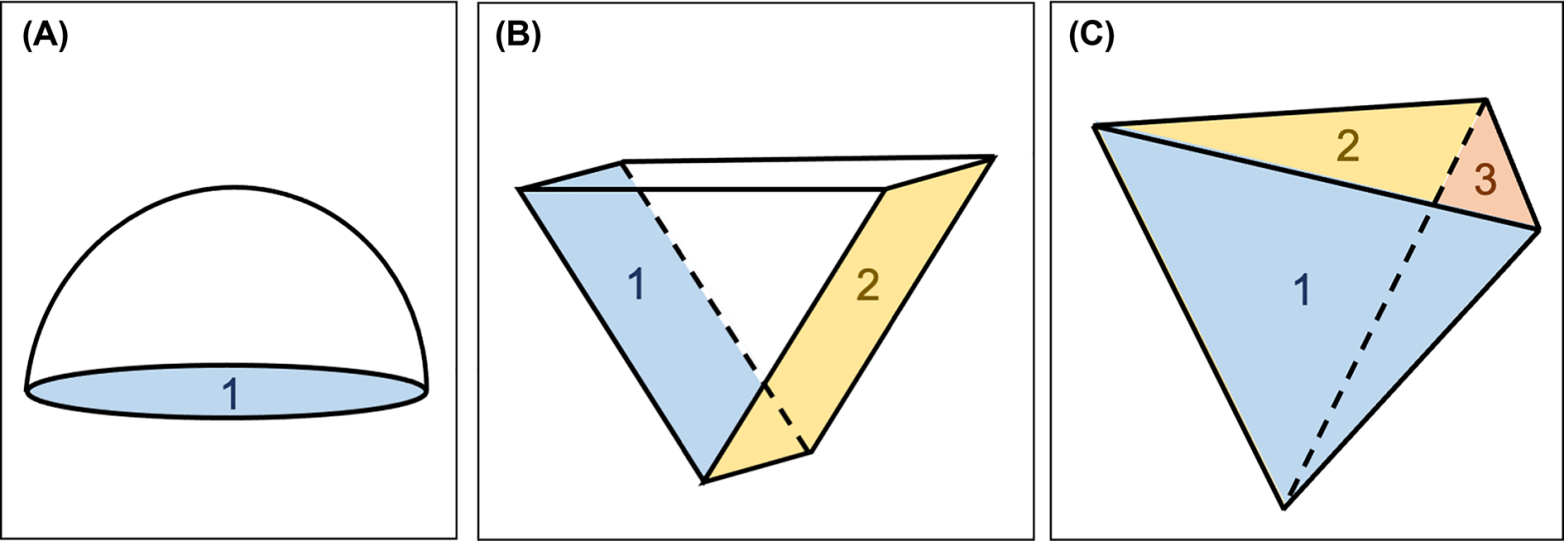
1D, 2D and 3D apical cells (**A**) Schematic diagram of an apical cell with one cutting face (1). (**B**) Schematic diagram of an apical cell with two cutting faces (1, 2). (**C**) Schematic diagram of a shoot tetrahedral apical cell of *P. patens* with three cutting faces; daughter cells are cut off from three rear faces (1, 2, 3) but never the shootward face that caps the shoot apex.

**Figure 2:**
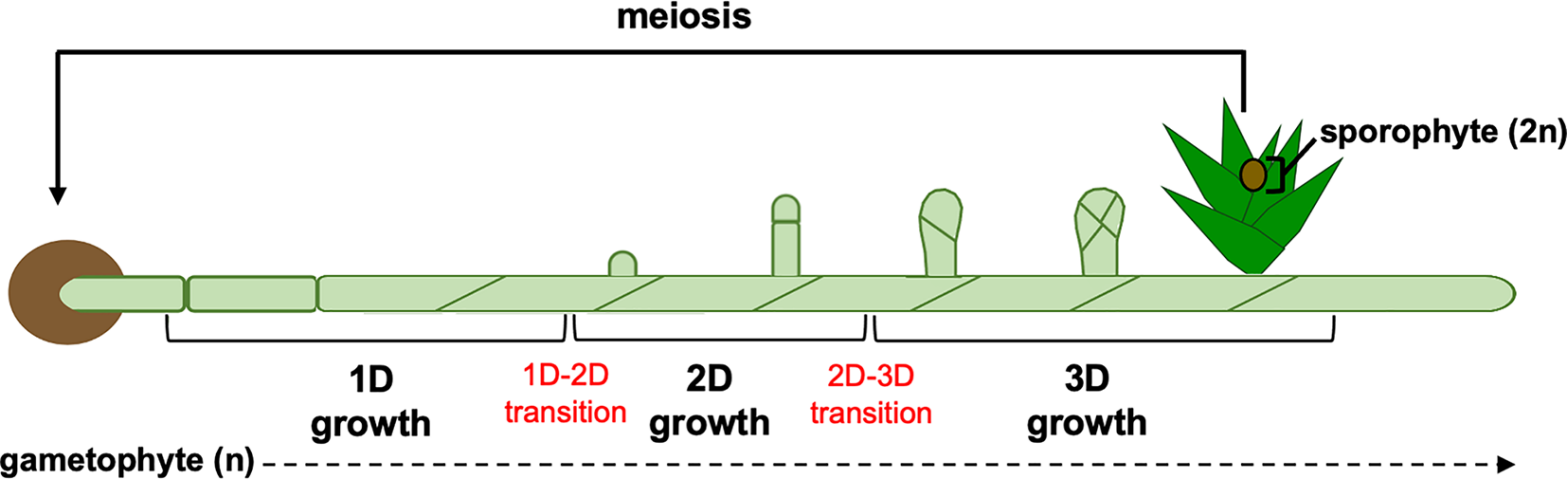
The life cycle of *P. patens* A haploid (n) spore germinates to form chloronemal cells. A chloronemal apical stem cell transitions into a caulonemal apical stem cell (shown by change in division angle), which forms filaments of caulonemal cells (1D). Side branch initials form that can give rise to either filaments (2D) or gametophores (3D). A characteristic oblique division distinguishes a filament initial from a gametophore initial. A regimented series of cell divisions leads to the formation of a gametophore with leaf-like phyllids arranged in a spiral phyllotaxy. Fertilization of an egg by motile sperm leads to the formation of the diploid sporophyte, which undergoes meiosis to produce haploid spores to restart the life cycle.

In the absence of cell movement, as in animals, it is the orientation of cell divisions combined with cell growth processes that form the major determinants of plant morphogenesis [3,4]. In land plants, growth is driven by apical stem cells (apical cells) that can divide to both self-renew and to produce distinct cell types. The geometry of the apical cell, and the way it divides, greatly influences the pattern of growth and development that follows. Some lineages of water-dwelling charophyte algae, from which land plants emerged, form apical cells but these are constrained to either 1D growth (apical cells have a single cutting face) (Figure 1A) or 2D growth (apical cells have a maximum of two cutting faces) (Figure 1B). Consequently, charophytes form simple body plans that commonly comprise filaments and mats [5,6]. An invariable feature of land plants is the ability to form and maintain apical cells with three or more cutting faces, a developmental innovation that paved the way to morphological diversity. To maximize light capture above ground, a single tetrahedral apical cell can give rise to shoots with lateral organs organized along or around a central axis (phyllotaxis), a mode of development typically observed in the gametophytes of mosses (‘spiral’) and leafy liverworts (‘three-ranked’), and the multicellular sporophytes of ferns (‘spiral’) and horsetails (‘whorled’) [7]. In this context, the tetrahedral apical cell resembles an inverted pyramid (Figure 1C). Below ground, the emergence of apical cells with three or more cutting faces enabled the elaboration of rooting structures in vascular plants [8,9]. By acquiring apical cells with three or more cutting faces, plants were able to develop the morphological complexity required to survive on land and to colonize progressively drier habitats.

Most plants grow in ways that make genetic studies of the transition from 2D to 3D growth difficult. This is because 3D growth begins during early embryonic development and disrupting 3D growth inevitably leads to the death of these plants. The moss *Physcomitrium patens* does not need to initiate 3D growth to survive and undergoes an extended 2D filamentous growth phase that can be cultured indefinitely. Consequently, insights into the mechanisms underpinning the formation of apical cells and the subsequent establishment and maintenance of 3D growth have largely been gained through studies in *P. patens*. This review summarizes recently published articles that have provided new and important insights into the mechanisms underpinning 3D growth in *P. patens*.

## 3D growth initiation, establishment and maintenance in *P. patens*

*Physcomitrium patens* is predominantly haploid and has a dimorphic gametophyte generation that comprises an extended 2D filamentous stage (protonemata) and a 3D shoot producing stage (gametophores). The life cycle of *P. patens* begins with the germination of haploid spores and the emergence of the tip growing chloronemal filaments. These exhibit 1D growth during filament extension until a chloronemal apical stem cell transitions into a caulonemal apical stem cell. Caulonemal filaments also extend by tip growth and form side branch initials (2D growth) that give rise to either chloronemal apical cells, caulonemal apical cells, or begin the 3D growth trajectory by forming gametophore apical cells (∼5%). Gametophore apical cells swell by probable diffuse growth and can initiate a highly coordinated cell division programme that establishes a tetrahedral apical cell that can both self-renew and rotate divisions through multiple planes. Consistently, daughter cells are cut off from the three downward faces of the apical cell, which resembles an inverted pyramid, but never the shootward face that caps the shoot apex (Figure 1C). By rotating cell division planes each time an apical cell divides, leaf-like phyllids can be arranged in a spiral-like manner around the central axis of the gametophore (spiral phyllotaxis). Gametophores bear both sperm-producing antheridia and the egg-producing archegonia; fertilization of egg cells by motile and biflagellated sperm cells leads to the formation of the diploid sporophyte, which undergoes meiosis to produce haploid spores to restart the life cycle [10–14] (Figure 2).

**Figure 3:**
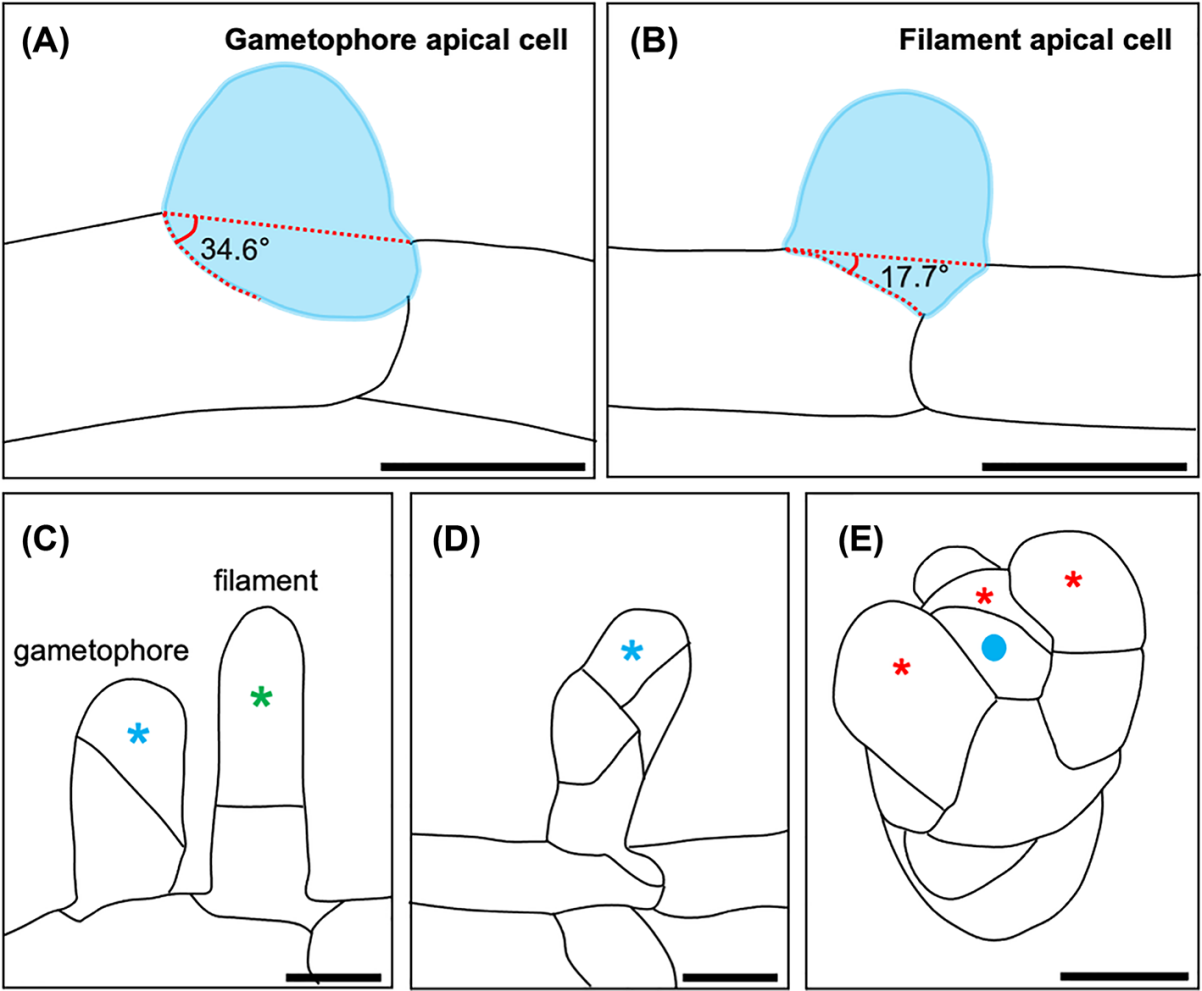
3D growth in *P. patens* (**A,B**) Schematic diagrams of the transition division leading to the formation of a gametophore apical cell (A) or a filament apical cell (**B**). (**C**) A gametophore apical cell (left) versus a filament apical cell (right). A filament apical cell divides parallel to the parental caulonemal cell from which it is derived, and tip growth is directed from apical cells (green asterisk). The first division of the gametophore apical cell is always oblique and leads to the formation of an apical cell (blue asterisk) and a basal cell. Subsequent divisions of both the apical cell and the basal cell occur obliquely to the first division (**D**). Additional rotating divisions establish a tetrahedral apical cell (blue dot) that divides to both self-renew and produce phyllid apical cells (red asterisks) that direct growth of phyllids (**E**). Scale bars represent 20 µm. Images adapted from [12,14].

There are three distinct phases of 3D growth in *P. patens*: (i) initiation – the formation of a gametophore apical cell, (ii) establishment – the cell division programme that leads to the development of a tetrahedral apical cell and (iii) maintenance – self-renewal of the apical cell once it is formed, and the continual generation of new daughter cells (merophytes) [12]. Gametophore apical cells are distinguishable from side branch initials that go on to form filaments. The first reliable predictor of 2D versus 3D cell fate is the discernible transition division that occurs; the division plane angle is greater in gametophore apical cells than in filament initials (Figure 3A,B). Interestingly, both 2D and 3D apical cells can form from the same parental caulonemal cell, which implies that the cues that dictate cell fate are highly localized. It has been suggested that local and transient polarizing signals may underpin these developmental decisions, although the underlying mechanisms remain poorly understood [12,14]. The first division of the gametophore apical cell is consistently oblique at the point at which 3D cell fate is irreversibly committed (Figure 3C). The apical and basal cells of the bud then divide obliquely and perpendicularly to the first division plane (Figure 3D). Two additional divisions of the apical cell permit the formation of a tetrahedral shaped apical cell, which in turn denotes the establishment of 3D growth. The apical cell is maintained throughout the lifetime of the plant to provide all the cell types required to produce a gametophore, notably by generating phyllid apical cells [12] (Figure 3E).

## Auxin and cytokinin

Gametophore apical cell formation can be induced by cytokinin and suppressed by high levels of auxin [15,16]. However, cytokinin alone is not sufficient to establish and maintain 3D growth, as exogenous cytokinin arrests gametophore development. Moreover, the cell-specific activity of cytokinin oxidase genes within developing buds implies that gametophore apical cell formation is immediately followed by some level of cytokinin degradation [17]. Exogenous auxin has the capacity to induce gametophore development in mutants that are cytokinin-resistant, demonstrating that cytokinin-mediated gametophore induction is dependent on auxin [15].

Auxin has been consistently implicated in cell division plane orientation within a broad range of developmental contexts, including the establishment of asymmetry in the embryo [18]. Recent evidence implicates auxin as the ‘instructive signal’ for the correct positioning of division planes in developing gametophores as the presence of auxin is particularly striking during the first three formative divisions of the bud. Furthermore, it appears that cell-type specific auxin accumulation occurs during bud development; notably auxin accumulation diminishes in many cells after the 4-cell stage and is strongly suppressed within the tetrahedral apical cell. As such, it appears that low auxin levels need to be sustained in the tetrahedral apical cell to maintain apical cell identity [19]. Cell fate decisions during 3D growth specification and shoot patterning (‘phyllotaxy’) are thus likely determined by distinct auxin and cytokinin distribution patterns in individual cell types [15,20,21].

## The cytoskeleton

**Figure 4:**
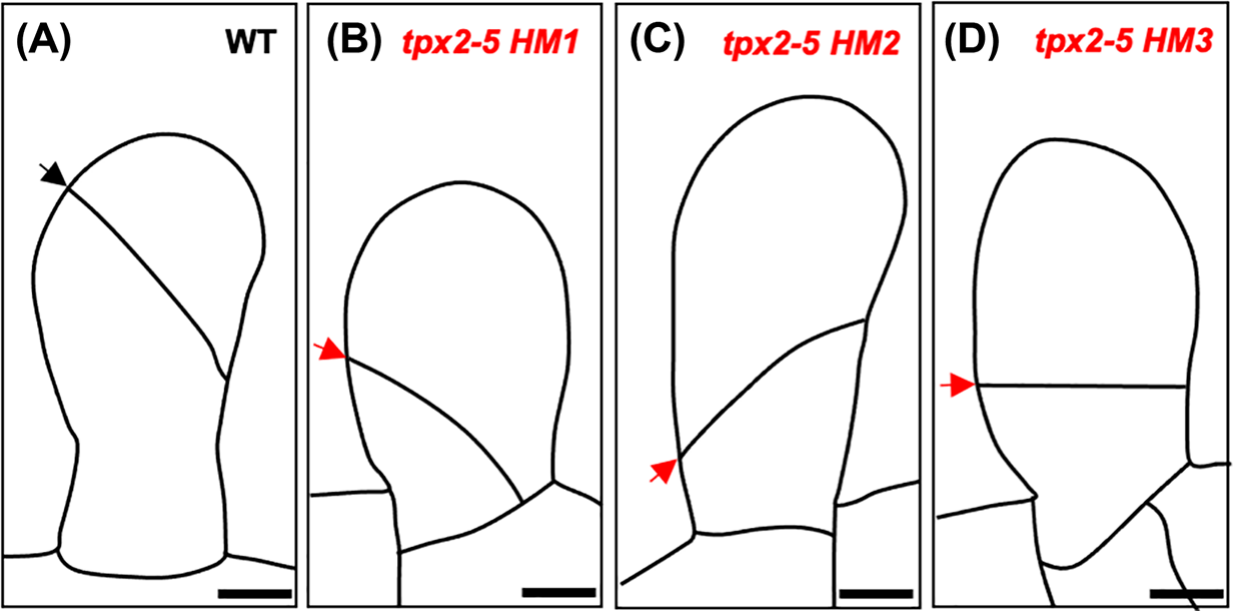
The hypomorphic *tpx2-5* mutants Schematic diagrams of the first division of the gametophore apical cell in wild-type (**A**) and incorrectly oriented divisions of the gametophore apical cell in the hypomorphic mutants *tpx2-5 HM1* (**B**), *tpx2-5 HM2* (**C**) and *tpx2-5 HM3* (**D**). Correctly and incorrectly oriented division planes are denoted by black and red arrows respectively. Scale bars represent 10 µm. Images adapted from [25].

Unlike animals, division sites within plant cells are pre-determined before mitosis [22,23]. In *P. patens*, the first three formative divisions of the bud are facilitated by microtubules independently of preprophase band (PPB) formation, and spindle orientation is guided by a transient acentrosomal microtubule organising centre known as the gametosome [24]. During PPB-independent cell division, the microtubule-associated protein targeting factor for Xklp2 (TPX2) plays an important role in determining the position of the division site. In the hypomorphic *tpx2-5* mutants, the first division of the gametophore apical cell is misdirected, and cell plates are positioned toward the base of cells (Figure 4). Consequently, mutants produce dwarf gametophores comprising smaller phyllids that exhibit cell proliferation defects. Treatment with latrunculin A, a reagent intended to depolymerize actin filaments, corrects the first characteristically oblique first division in these mutants. Thus, the spindle positioning defects observed in *tpx2-5* mutants have been attributed to actin microfilaments [25].

It is the PPB that dictates division plane orientation during the latter stages of gametophore development. Formation of the PPB is dependent on TONNEAU1, a feature that is evolutionarily conserved throughout the land plants [26]. In *P. patens* mutants lacking TONNEAU1 function, PPBs are not formed, cell division planes are randomly oriented, and cellular elongation defects severely affect gametophore development [24,26]. Notably, TONNEAU1 function has been linked to auxin signaling in flowering plants [27], suggesting that auxin-mediated cell division plane orientation is a conserved and fundamental feature of plants.

## Regulators of gametophore apical cell formation

The APB transcription factors are among the master regulators of gametophore apical cell formation; mutants that lack all four *APB* genes (*PpAPB1–4*) fail to form gametophores, even in the presence of cytokinin [13]. It has been proposed that NO GAMETOPHORES 1 (PpNOG1), which contains a prominent ubiquitin-associated domain, may degrade a repressor of *APB* gene transcription via proteasome-mediated degradation. This is because mutants that lack a functional copy of PpNOG1 produce fewer gametophore apical cells than wild-type and these mutants are also cytokinin unresponsive. The first oblique division of the gametophore apical cell is also misoriented in these mutants, preventing the establishment of a tetrahedral apical cell [28]. Thus, the APBs and NOG1 represent positive regulators of gametophore apical cell formation, along with Mycorrhizae-like PpMACRO2 [29] and the glycerol-3-phosphate acyltransferases PpGPAT2 and PpGPAT4 [30]; mutants lacking these genes also exhibit a strong reduction in gametophore apical cell formation. Furthermore, it has been shown that, when key components of the Polycomb Repressive Complex 2 (PRC2) are disrupted, 3D growth is prevented by the ectopic formation of apogamous sporophytes [31–33]. Several negative regulators of gametophore apical cell formation have also recently been reported, including DEFECTIVE KERNEL 1 (DEK1) [34–37], CLAVATA [38,39], NO GAMETOPHORES 2 (PpNOG2) [40] – also identified as HYDROXYCINNAMOYL-COA:SHIKIMATE HYDROXYCINNAMOYL TRANSFERASE (PpHCT) [41], and the exocyst subunit PpSEC6 [42]. Mutants lacking these regulators not only produce supernumerary gametophore apical cells but also exhibit severe defects in division plane orientation at the earliest stages of gametophore development. We also understand a little about the genetic toolkit required to maintain a tetrahedral apical cell [43]. The following sections highlight recent progress in the field.

## Polycomb repressive complex 2

The PRC2 is a multi-subunit protein complex that regulates gene repression by facilitating histone tri-methylation (H3K27me3). In Arabidopsis, loss of either of the PRC2 components CURLY LEAF (CLF) or FERTILIZATION-INDEPENDENT ENDOSPERM (FIE) leads to loss of tri-methylation and significant disruption to developmental processes [44–46]. In *P*. patens, it has been demonstrated that CLF and FIE proteins can interact, and disruption of either of the respective genes can alter the fate of side branch initials; mutants produce a large abundance of sporophyte-like apical cells that give rise to cone-shaped sporophyte-like bodies in the haploid gametophyte. Genes that are ordinarily only expressed in the sporophyte of wild-type are expressed in these sporophyte-like bodies, including *MKN2*, *MKN4* and *MKN5* [31,32,47]. Strikingly, loss of tri-methylation in these mutants is strongly associated with the enhanced accumulation of *PpBELL1* and *PpBELL2* transcripts [31,33]. It has also been demonstrated that ectopic expression of *PpBELL1* can drive embryo formation from caulonemal filaments in the absence of fertilization, but disrupting *PpBELL1* function in *PpFIE* deletion lines can restore normal 3D growth [48]. Thus, the PRC2 complex is required to promote the formation of gametophyte apical cells and block the formation of apogamous sporophytes. In the absence of a functional PRC2 complex, gametophore apical cells cannot form, and 3D growth cannot occur.

## Defective Kernel 1 (PpDEK1) and the SOSEKI proteins

Defective Kernel 1 (PpDEK1) is a membrane-bound protease that is only found within land plants. The protein comprises two transmembrane regions that are separated by a Loop domain (collectively the membrane segment), and a Linker region that connects the membrane segment to a catalytic calpain domain that is released into the cytosol following an intramolecular cleavage event [34–36]. In mutants lacking PpDEK1 function, supernumerary gametophore apical cells form. Furthermore, mutants also exhibit division plane orientation defects early during gametophore development and fail to establish a tetrahedral apical cell [34]. Previously, it was proposed that PpDEK1 antagonizes PpNOG1 function to regulate gametophore apical cell formation, by degrading an activator of *APB* gene transcription [28]. To support this proposition, *APB* gene transcripts accumulate to excessive levels in *Ppdek1* deletion mutants, and *PpDEK1* overexpression results in delayed gametophore initiation [34,35].

**Figure 5:**
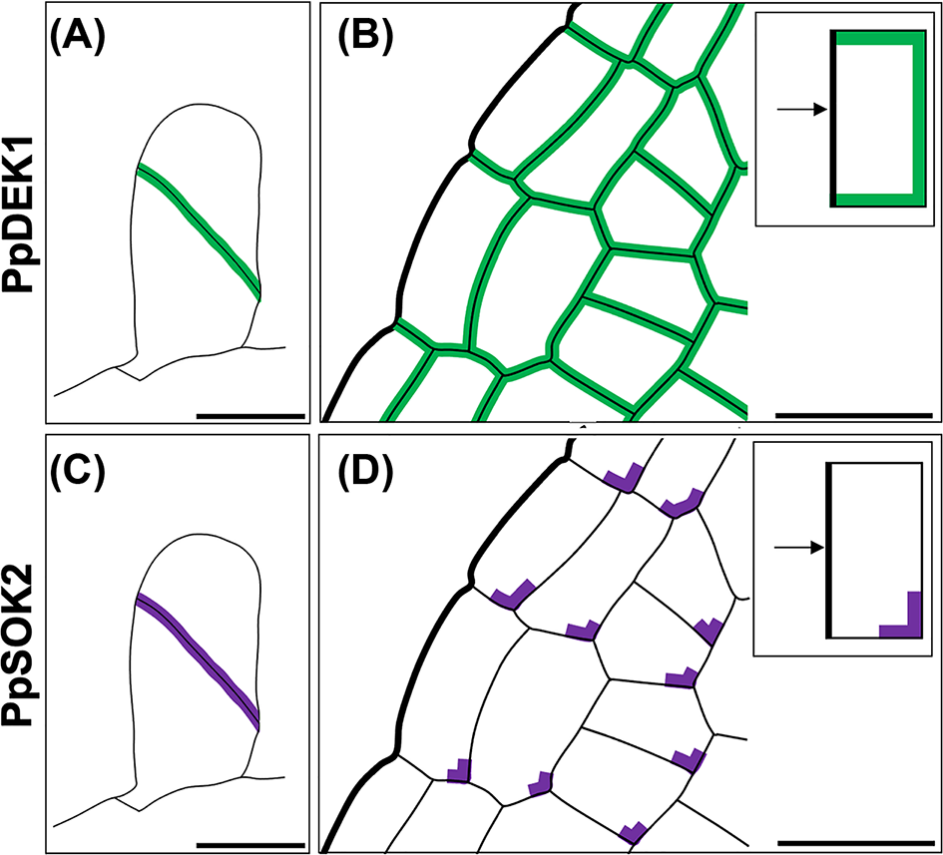
Polar localization of PpDEK1 and PpSOK2 (**A, B**) Polar localization of PpDEK1 following the first division of the gametophore apical cell (A) and in developing phyllids (B). Inset in (B) shows a notable absence of PpDEK1 localization at phyllid margins. (**C, D**) Polar localization of PpSOK2 following the first division of the gametophore apical cell (C) and in developing phyllids (D). Inset in (D) highlights that PpSOK2 is localized at the base of cells within the phyllids and is enriched in the corners that are more distal to the phyllid margins. Scale bars represent 20 µm. Images adapted from [37,51].

**Figure 6:**
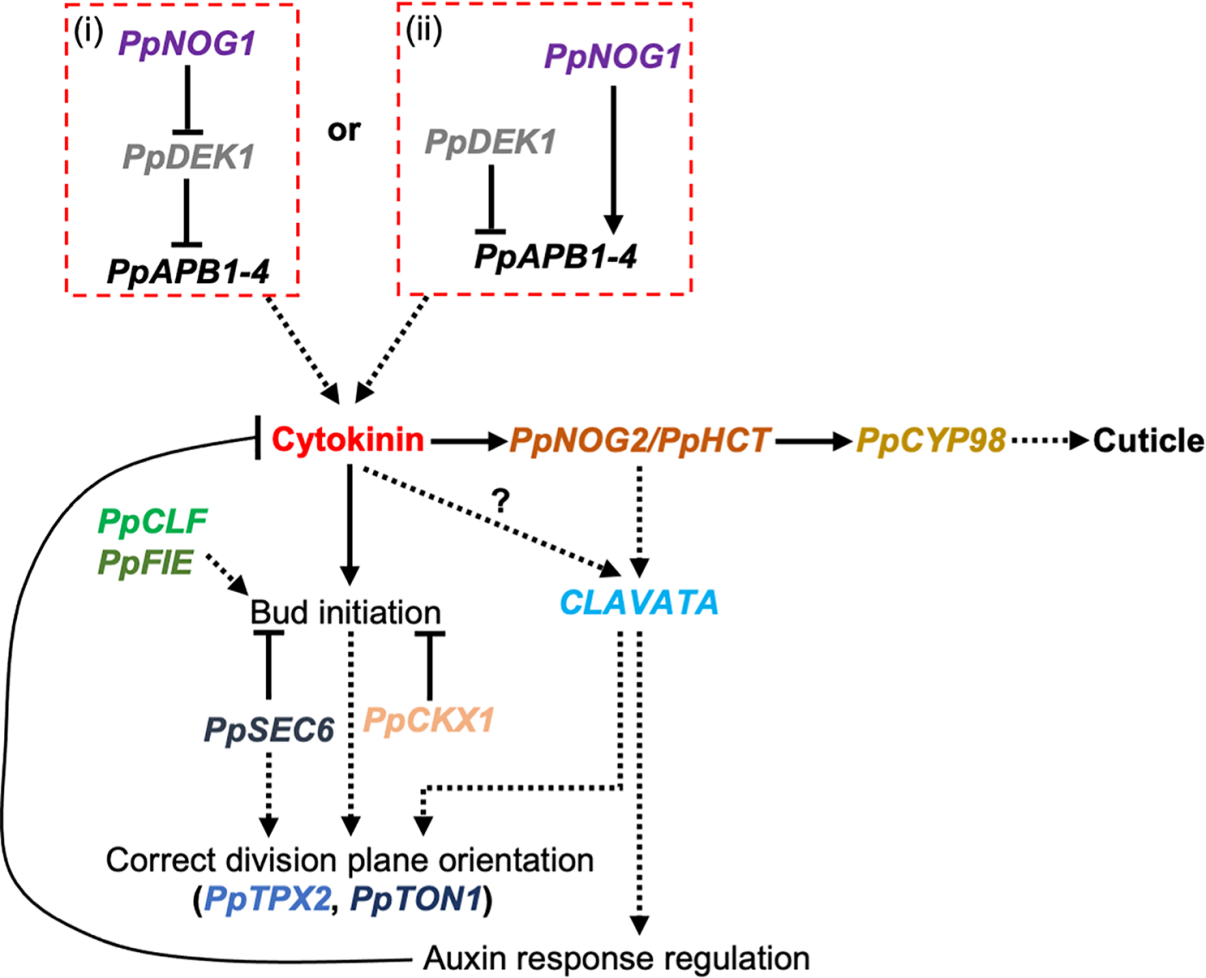
Proposed model for 3D growth in *P. patens* Two possible modes of *PpAPB* gene regulation have been proposed: (i) PpNOG1 may target PpDEK1 for proteasome-mediated degradation to relieve the repression of *PpAPB* gene transcription [37]; or (ii) PpNOG1 and PpDEK1 operate independently and antagonistically to degrade a repressor and activator of *PpAPB* transcription, respectively [40]. In either case, PpAPBs are likely to promote cytokinin biosynthesis, which in turn initiates gametophore apical cell formation. High levels of cytokinin induce *PpHCT*/*PpNOG2* expression, which activates a CLAVATA-dependent auxin response to suppress the cytokinin response and prevent supernumerary gametophore apical cells from being formed. The correct balancing of auxin and cytokinin levels also ensures that division planes within developing gametophores are positioned correctly, a process that is dependent on microtubules, PpTPX2, and subsequently PpTON1. This can also be achieved through concerted assistance from PpCKX1 (which drives local cytokinin degradation). PpCLF and PpFIE are required to maintain the identity of gametophyte apical cells and are thus key regulators of 3D growth. The exocyst probably establishes polarity cues that both dictate apical cell fate and orient the mitotic spindle.

It has since been demonstrated that PpDEK1 exhibits a striking subcellular polarity during the three distinct phases of 3D growth, and that this localization is independent of calpain domain proteolytic activity. PpDEK1 is abundantly localized to the plasma membrane that lies at the interface between the most recently divided and/or elongating cells, from the first oblique division of the gametophore apical cell (Figure 5A) through to development of the phyllids (Figure 5B). The authors present an alternative but plausible model for gametophore apical cell formation, suggesting that PpDEK1 is targeted for degradation by PpNOG1. They propose that, when associated with newly formed cell walls, PpDEK1 is stabilized by currently undefined stabilizing signals and protected from PpNOG1-mediated degradation. Any PpDEK1 that is not associated with newly formed cell walls would however be susceptible to PpNOG1-mediated degradation, leading to polarization of the PpDEK1 protein [37]. Similarly, to the previously proposed model [28], this model can explain differences in gametophore apical cell number in *Ppnog1R* [28] versus *Ppdek1* [34–37] mutants but does not explain why division plane orientation is defective. The detected PpDEK1 localization patterns are reminiscent of those reported for the PIN proteins. However, the expression of PIN proteins is not evident at the earliest stages of bud development and persists in mature phyllids long after PpDEK1 expression fades [49,50]. Thus, it is likely that alternative factors govern PpDEK1 polarity. One possibility is the recently described SOSEKI proteins, two of which have been shown to display polar localization patterns: PpSOK4 is polar localized in caulonemal filaments and following the switch to 3D growth whereas PpSOK2 expression is constrained to developing gametophores – PpSOK2 localizes at newly formed cell plates of early buds (Figure 5C) and is corner localized in cells of developing phyllids, particularly those located toward the base (Figure 5D) [51]. SOSEKI proteins recruit ANGUSTIFOLIA (AN) to polar sites in Arabidopsis roots, which regulate cell division and growth respectively [51–53]. In *P. patens*, AN regulates diffuse growth within developing gametophores but does not play a role in cell division orientation [54,55]. The function of *P. patens* SOSEKI proteins remains unclear, but since ‘breaking symmetry’ is considered a prerequisite for the establishment of a tetrahedral apical cell, we could speculate that SOSEKI proteins regulate this process. If this is the case, disrupting SOSEKI function is likely to perturb 3D growth (Figure 6).

## CLAVATA

CLAVATA signalling components are found extensively throughout the land plants. In *P. patens*, these comprise two CLAVATA1-like receptors (PpCLV1a and PpCLV1b), receptor-like protein kinase 2 (PpRPK2) and four CLAVATA3-like peptides that are collectively encoded by nine genes (*PpCLE1-9*); *PpCLE1*, *PpCLE2*, *PpCLE3*, *PpCLE8* and *PpCLE9* encode the same CLE peptide; *PpCLE5* and *PpCLE6* encode the same CLE peptide; and *PpCLE4* and *PpCLE7* each encode distinct CLE peptides [38,56]. Significantly, CLAVATA regulates hormone homeostasis to modulate the activity of apical cells and has been linked to both auxin [56] and cytokinin signalling [39].

Disruption of CLAVATA leads to highly pronounced defects in the establishment and maintenance of 3D growth. Notably, cell division planes are misoriented in emerging buds and supernumerary apical cells are formed along the swollen stems of mutant gametophores [38]. In triple mutants that lack functional copies of all three receptors (*clv1a/clv1b/rpk2*), ectopic apical cells form, all of which can initiate phyllids. Ectopic apical cell formation can be phenocopied in wild-type by treating gametophores with moderate levels of exogenous cytokinin, suggesting cross-talk between CLAVATA and cytokinin signalling pathways. However, ectopic apical cells also form in mutants lacking the core components of cytokinin perception, namely the CHASE domain-containing histidine kinase receptors (*chk1 chk2 chk3*). This presents somewhat of a conundrum, that the authors reason could be due to incoherent feedforward control [39]. Nevertheless, CLAVATA receptors possess a ‘stem cell-delimiting’ function that appears to be highly conserved [38,39].

## The cuticle (NOG2/CYP98/GPAT)

The cuticle was one of the most important evolutionary adaptations that enabled terrestrialization, forming an effective barrier against water loss and pathogen attack, and serving to delineate organ boundaries. In seed plants, permeability of the cuticle is controlled by the biopolymers cutin, suberin and lignin. The *P. patens* cuticle contains cutin and cuticular waxes but is largely considered to be non-lignified and non-suberised [30]. Instead, a pre-lignin pathway enables the formation of a cuticle in gametophores, providing rigidity to support upright growth; a cuticle is not formed in the protonema [30,41,57]. Glycerol-3-phosphate acyltransferases (GPAT) provide precursors of cutin biosynthesis; In mutants lacking a functional copy of the *PpGPAT2* gene, fewer gametophores are formed, and water loss accelerates due to enhanced cuticle permeability [30].

In *P. patens*, PpCYP98 (C3′H) and hydroxycinnamoyl-coenzyme A (CoA):shikimate hydroxycinnamoyl transferase (PpHCT) act within an ascorbic acid/hydroxycinnamoyl-threonate pathway leading to the formation of a pre-lignin cuticle biopolymer. These components were likely co-opted into the lignin biosynthesis pathway following the divergence of bryophytes and vascular plants. PpHCT transfers alcohols or amines to the substrate p-coumaroyl CoA, which acts as a precursor for both phenylpropanoid (‘the cuticle’) and flavonoid biosynthetic pathways, to form p-coumaroyl threonate. PpCYP98 then catalyzes the downstream step in the pathway. Mutants lacking a functional copy of either the single copy genes *PpCYP98* or *HCT* (also called *PpNOG2*) fail to develop gametophores and exhibit defects in cuticle biopolymer formation [40,41,57]. Notably, the caffeate precursors required for cuticular biopolymer formation are absent in these mutants [41,57]. Despite residing in distinct pathways, *PpHCT/PpNOG2* can complement for the loss of Arabidopsis *HCT* [41]. Loss of *PpHCT*/*PpNOG2* affects apical cell fate; supernumerary gametophore apical cells are formed, and emerging buds exhibit an array of developmental defects. Two different developmental trajectories have been reported in *P. patens*: (1) the first two divisions of the bud occur normally but the third cell division plane is misplaced and leads to early developmental arrest (defective 3D growth); (2) after the second division of the bud, the cell intended to form a rhizoid (the basal lateral cell) enlarges and concurrently, alongside the intended apical cell, enters a cell division programme that causes early developmental arrest. Defects are attributed to the presence of reduced levels of endogenous auxin within these mutants. *PpHCT*/*PpNOG2* is strongly induced by cytokinin, and therefore may play a role in a feedback loop designed to prevent ectopic gametophore apical cell formation [40].

## The exocyst

The exocyst mediates the tethering process during exocytosis, and represents an octameric protein complex comprising SEC3, SEC5, SEC6, SEC8, SEC10, SEC15, EXO70 and EXO84 subunits. When the formation of the exocyst is disrupted in *P. patens*, gametophore apical cells can form from both caulonemata and chloronemata [42,58]. In *Ppsec6* mutants, supernumerary gametophore apical cells are formed, and striking cell division defects are evident throughout development. The presence of cell wall stubs and multinucleated cells demonstrates that incomplete cytokinesis can occur in both protonemata, and in developing gametophores. The division programme of the bud also deviates from the norm from the fourth cell division onwards, a tetrahedral apical cell cannot form and development of the gametophore arrests. It is known that the secretory pathway cooperates with the cytoskeleton to regulate plant growth and development [42]. It is therefore likely that disruption of polarity cues, and/or the localization of the mitotic spindle is responsible for the perturbations observed in these mutants.

## Angustifolia3

Angustifolia3 (AN3) is homologous to the human transcription coactivator synovial sarcoma translocation protein (SYT) [59] and has been shown to promote cell proliferation in the leaves of flowering plants [60]. The *P. patens* genome encodes four AN3-related genes, *PpAN3-1* to *PpAN3-4*, that are abundantly expressed in gametophores. Single and sequentially generated double, triple, and quadruple loss-of-function mutants showed progressive and gene-dosage dependent defects in gametophore development. Quadruple mutants form conspicuously stunted gametophores that form fewer phyllids, each comprising fewer cells than wild-type. Although tetrahedral apical cells can form in these mutants, the reduced proliferative activity of the tetrahedral apical cell caused fewer phyllid apical cells to develop. Arginine levels are elevated in these mutants, and it is possible to phenocopy mutants by treating wild type with exogenous arginine. Thus, arginine maintains the tetrahedral apical cell during gametophore development, downstream of the *AN3* genes [43].

## Summary

- 3D growth in *P. patens* is regulated by a complex toolkit that operates on many levels; epigenetically [31–33], transcriptionally [13] and post-translationally [28,31–37,40,41].
- The formation of gametophore apical cells is underpinned by complex auxin–cytokinin cross-talk, the nature of which remains rather poorly understood.
- When the fine balancing act between auxin and cytokinin is disturbed, there is a significant impact on the types of apical cells that form and/or the way they divide.
- Although models of 3D growth continue to be described, we are still missing several pieces of the puzzle. Continued efforts within the field, using a combination of forward and reverse genetics studies, will help to resolve these models.

## Open Access

Open access for this article was enabled by the participation of University of Oxford in an all-inclusive *Read & Publish* agreement with Portland Press and the Biochemical Society under a transformative agreement with JISC.

## Abbreviations

AN3: Angustifolia3
CLF: CURLY LEAF
FIE: FERTILIZATION-INDEPENDENT ENDOSPERM
GPAT: Glycerol-3-phosphate acyltransferases
PPB: preprophase band
PRC2: Polycomb Repressive Complex 2
